# Discovering the Mutational Profile of Early Colorectal Lesions: A Translational Impact

**DOI:** 10.3390/cancers13092081

**Published:** 2021-04-25

**Authors:** Chiara Alquati, Anna Prossomariti, Giulia Piazzi, Francesco Buttitta, Franco Bazzoli, Luigi Laghi, Luigi Ricciardiello

**Affiliations:** 1 Department of Medical and Surgical Sciences, University of Bologna, 40138 Bologna, Italy; chiara.alquati2@unibo.it (C.A.); anna.prossomariti2@unibo.it (A.P.); giulia.piazzi@unibo.it (G.P.); francesco.buttitta@unibo.it (F.B.); franco.bazzoli@unibo.it (F.B.); 2 Center for Applied Biomedical Research (CRBA), University of Bologna, 40138 Bologna, Italy; 3 Department of Medicine and Surgery, University of Parma, 43126 Parma, Italy; luigiandreagiuseppe.laghi@unipr.it; 4 Laboratory of Molecular Gastroenterology, IRCCS Humanitas Clinical and Research Center, 20089 Rozzano, Italy

**Keywords:** colorectal cancer, familial adenomatous polyposis, lynch syndrome, conventional colorectal adenomas, serrated colorectal adenomas

## Abstract

**Simple Summary:**

Colorectal cancer (CRC) is one of the most common malignancies worldwide. Next-generation sequencing technologies have identified new candidate genes and deepened the knowledge of the molecular mechanisms underlying the progression of colonic adenomas towards CRC. The main genetic, epigenetic, and molecular alterations driving the onset and progression of CRC in both hereditary and sporadic settings have also been investigated. The evaluation of the CRC risk based on the molecular characterization of early pre-cancerous lesions may contribute to the development of targeted preventive strategies development, help define specific risk profiles, and identify patients who will benefit from targeted endoscopic surveillance.

**Abstract:**

Colorectal cancer (CRC) develops through a multi-step process characterized by the acquisition of multiple somatic mutations in oncogenes and tumor-suppressor genes, epigenetic alterations and genomic instability. These events lead to the progression from precancerous lesions to advanced carcinomas. This process requires several years in a sporadic setting, while occurring at an early age and or faster in patients affected by hereditary CRC-predisposing syndromes. Since advanced CRC is largely untreatable or unresponsive to standard or targeted therapies, the endoscopic treatment of colonic lesions remains the most efficient CRC-preventive strategy. In this review, we discuss recent studies that have assessed the genetic alterations in early colorectal lesions in both hereditary and sporadic settings. Establishing the genetic profile of early colorectal lesions is a critical goal in the development of risk-based preventive strategies.

## 1. Introduction

In approximately 75–80% of cases, colorectal cancer (CRC) occurs sporadically, without a genetic predisposition. At least 10–20% of CRC cases have a positive family history, and up to 5–7% of cases develop as a consequence of hereditary CRC-predisposing syndromes [1]. In 1988, Vogelstein and colleagues defined the principles of the adenoma-carcinoma sequence, also known as the “conventional” CRC pathway and first described the molecular bases of CRC evolution. Most CRC cases arise from conventional colorectal adenomas (CNADs) [2]. CNADs (including tubular, tubulovillous, and villous adenomas) are polypoid lesions of the colonic mucosa which, in some cases, evolve into CRC [3]. Subsequently, another unconventional pathway was described in the pathogenesis of CRC, also known as the “serrated” pathway [4]. Approximately 15–30% of CRCs develop from serrated lesions [5,6,7]. The World Health Organization has classified colorectal serrated lesions as: (i) hyperplastic polyps (HPs), (ii) sessile serrated lesions (SSLs), previously classified as sessile serrated adenomas, (iii) SSLs with dysplasia, (iv) traditional serrated adenomas (TSAs), and (v) unclassified serrated adenomas [8,9]. Traditionally, SSLs and TSAs are pre-cancerous colorectal lesions, while HPs mainly have a low risk of developing CRC.

Several studies have prospectively analyzed the long-term CRC risk in patients with CNADs or serrated lesions and found an increased CRC risk in patients with advanced high-risk adenomas or large serrated polyps [10,11,12,13,14,15]. A recent study on CRC incidence and mortality in a large cohort of fecal occult blood test positive subjects showed that patients with colorectal neoplastic lesions, regardless of subtype, have an increased risk of developing CRC. The mortality for CRC was higher in patients with SSLs, tubulovillous adenomas, and villous adenomas [16].

The malignant transformation of normal colonic mucosa towards adenoma and then CRC requires the accumulation of genetic and epigenetic alterations. These events occur over an average time span of up to 15 years in the conventional pathway [1]. The genetic drift, however, accelerates these events in hereditary syndromes, implying an early and restricted time frame for surveillance [17]. CRC development through the serrated pathway would also seem to occur more rapidly than the conventional pathway [18].

Three main genetic pathways for CRC development have been described: (i) the chromosomal instability pathway (CIN, ~80% of CRC cases), frequently observed in the conventional pathway and distal CRC, driven by chromosomal alterations/rearrangements, and mutations in known oncogenes (*KRAS*, *BRAF*, *PIK3CA*) and tumor suppressor genes (*APC*, *SMAD4*, *TP53*); (ii) the CpG island methylator phenotype (CIMP, ~15–30% of CRC cases) due to a diffuse CpG island methylation mostly observed in proximal CRC and frequent in the serrated pathway associated with *BRAF* mutations; iii) the microsatellite instability pathway (MSI, ~15% of CRC cases), in which the genomic instability is driven by germline (Lynch syndrome—LS) or sporadic inactivation of the mismatch repair (MMR) genes (*MLH1*, *MSH2*, *MSH6*, and *PMS2*) [19]. Notably these pathways can overlap in CRC pathogenesis and also co-exist within the same tumor [20].

Hereditary CRC syndromes encompass (i) non-polyposis hereditary syndromes including LS, Lynch-like syndrome, familial CRC type X, (ii) polyposis syndromes including familial adenomatous polyposis (FAP), MUTYH-associated polyposis (MAP), and the serrated polyposis syndrome (SPS); and (iii) hamartomatous syndromes including Peutz-Jeghers syndrome, juvenile polyposis syndrome, and PTEN-hamartomatous tumor syndrome [17,21].

Yurgelun and colleagues recently evaluated the presence of germline mutations in a large cohort of unselected CRC patients. Interestingly, they found that approximately 10% of the patients had germline causative mutations in CRC-predisposing genes as well as in genes not conventionally associated with CRC, thus indicating that the percentage of CRC cases due to hereditary defects may be higher than generally thought [22]. Many studies have defined distinct mutational profiles of advanced CRCs [20,23,24] and four distinct consensus molecular subtypes (CMS1-CMS4) for CRC have been described [25] according to their patterns of mRNA expression.

In addition, the somatic mutational cancer signatures in CRC and colonic polyposis have been recently reviewed [26] and various studies have focused on the genetic and molecular characterization of pre-cancerous colonic lesions.

In this review, we discuss recent findings with regard to defining the somatic alterations of both hereditary and sporadic early colorectal lesions. We focus on FAP, MAP, and LS-derived adenomas as part of the hereditary setting, as well as conventional and serrated lesions in the sporadic setting.

## 2. Hereditary Settings

### 2.1. Familial Adenomatous Polyposis and MUTYH-Associated Polyposis

FAP is an inherited autosomal dominant syndrome clinically characterized by the onset of hundreds to thousands of adenomatous polyps in the gastrointestinal tract at a young age. Given that FAP patients are at high risk of CRC (almost 100% at a mean age of 39 years), they require close surveillance and endoscopic treatment for CRC prevention. However, most of them need undergoing prophylactic surgery to counteract the progression of polyps towards cancers [27]. Moreover, extra-intestinal clinical manifestations are common in FAP [27]. FAP arises from heterozygous germline mutations in the tumor suppressor *APC* gene, leading to aberrant Wnt/β-catenin pathway activation and sustained intestinal hyperproliferation [27]. Somatic mosaicism in the *APC* gene is also a frequent causative event for FAP [28], as well as in sporadic polyposis patients [29].

Unlike FAP, MAP is a hereditary autosomal recessive syndrome caused by germline mutations in the *MUTYH* gene. Genetic alterations in the *MUTYH* gene confer an increased CRC risk (from 43% to 100% at 48 years) due to the improper functioning of one of the DNA damage repair mechanisms. The clinical phenotype of MAP patients resembles attenuated FAP, typically presenting from 10 up to 100 colorectal polyps [30,31]. Although the genetic basis for these syndromes is known, many studies conducted in recent years have characterized the somatic alterations that cause the progression of adenomas in these patients (Table 1).

#### Somatic Mutational Profile in FAP and MAP

The somatic mutational profile of colorectal pre-cancerous lesions in FAP was recently investigated in a study on 25 colorectal adenomas and adjacent normal mucosa from 12 FAP patients with confirmed germline *APC* gene mutations through whole-exome sequencing (WES) [32]. This study confirmed the pivotal effect of Wnt signaling alterations in the early stages of colorectal carcinogenesis in these patients. In fact, a somatic second hit in the *APC* gene was found in most adenomas (72%) and a small fraction of sequenced adenomas also had further somatic mutations in Wnt signaling pathway components including *TCF7L2*. Damaging somatic mutations on *KRAS* and *FBXW7* genes also occurred, as well as potential deleterious mutations in additional genes including *CNOT3*, *EWSR1* and *PCMTD1* (Table 1). Allelic imbalance was also frequently observed in the analyzed adenomas and validated in an additional cohort of 37 adenomas from 14 FAP patients. In particular, the loss of 5q and amplifications in chromosomes 7 and 13 appeared to be relevant in the early phases of colonic transformation. The authors also showed an early development of intra-tumor heterogeneity (ITH) since most adenomas harbored sub-clones arising from an *APC*-mutant founder clone [32].

Multiple somatic inactivating mutations in the *APC* and *KRAS* genes have also been found in a study on 37 FAP-derived adenomas. The study provided further evidence about the early onset of ITH in colorectal tumorigenesis, demonstrating that *APC*-mutant adenomas have polyclonal characteristics with different mutations arising from independent lineages. Specifically, the analysis of individual colonic crypts isolated from adenomas of patients with distinct hereditary polyposis syndromes, as well as from sporadic and Lynch-syndrome derived carcinomas, demonstrated that the ITH for both *APC* and *KRAS* alterations was detectable in single crypts [33] (Table 1). These results suggest that multiple clones may compete for malignant transformation within the same pre-cancerous lesion.

Combining next-generation sequencing (NGS) with single-cells transcriptomic analysis, new data have been provided on the genetic and transcriptomic alterations occurring in the transition from adenomas to carcinoma in FAP individuals. The authors performed WES, whole-genome-sequencing (WGS) and single-cell-RNA-sequencing on normal mucosa, adenomas and carcinomas from five FAP patients and one patient with MAP. In line with previous studies, the authors showed that somatic inactivation of the *APC* gene constitutes the most frequent event in adenomas from FAP patients and that the derangements of Wnt signaling represented the most affected pathway. Considering all the samples analyzed (including normal mucosa) in this study from FAP patients and the single patient with MAP, the authors found that potential driver alterations in *TTN*, *SMAD4*, *GNAS*, *ASXL1*, *KRAS*, *FAT4*, *ZFHX3*, *FBXW7*, *PTPRT,* and *SOX9* genes occur with a frequency of between 37% and 16% (Table 1). In addition, by analyzing multiple adenomas from individual patients, it was found that different lesions collected from closely related colonic areas might originate from the same cell following a field cancerization process. By sequencing multiple regions within the same lesion, the authors observed ITH as well intertumoral heterogeneity in pre-cancerous lesions, confirming that both occur in the early phases of colonic carcinogenesis. Finally, morphologically normal colonic mucosa from FAP patients had a transcriptomic profile indicative of an early metabolic switch typical of cancer cells, particularly regarding the carbohydrate metabolism, as well as a hyperproliferative signature [34].

A characterization of the somatic mutational profile performed with WES of colorectal adenomas from FAP and MAP patients showed that adenomas from MAP patients had a higher rate of missense and nonsense mutations compared with FAP adenomas, due to genetic defects in the *MUTYH* gene. Moreover, *APC*, *KRAS,* and *WTX* (*AMER1*) genes were frequently mutated in pre-cancerous lesions from these patients. In particular, *APC* somatic mutations occurred in 50% of the tumors in this study [35] (Table 1). Mutations in the *WTX* gene, encoding for a negative regulator of Wnt/β-catenin signaling [39], have also been associated with sporadic CRC [40], and might contribute to CRC initiation [35].

A subsequent study employing WES analyzed the somatic mutational signature of multiple duodenal adenomas, which represent a frequent extra-colonic manifestation in these syndromes, from 16 FAP and 10 MAP patients, respectively [41]. Similarly to colorectal adenomas, duodenal adenomas from MAP patients also had an increased mutational burden compared with FAP adenomas. Most somatic mutations found in MAP adenomas were G>T transversions. Unlike colorectal adenomas, no mutations in the *WTX* gene were identified in duodenal adenomas. On the other hand, *APC* and *KRAS* have been found to be recurrently mutated also in duodenal adenomas. Moreover, *PTCH2*, *ERBB2* and *PCL1* genes have also been found to be potentially involved in duodenal carcinogenesis in these patients [41].

### 2.2. Lynch Syndrome

LS is the most frequent hereditary CRC-predisposing syndrome with an autosomal dominant pattern [42]. Germline mutations in the mismatch repair (MMR) genes, particularly *MLH1*, *MSH2*, *MSH6,* and *PMS2,* are causative for LS development together with *EPCAM* gene deletions which result in *MSH2* epigenetic silencing [42].

The inactivation of MMR genes thus represents the mechanism underlying LS tumors onset and progression [43]. However, the identification of small low-grade adenomas with intact MMR genes in these patients suggests that additional events and factors may be involved in LS-associated adenoma development [43,44,45,46]. Although MSI is the hallmark of LS-CRCs [45,47], data are conflicting on the precise timing of complete MMR inactivation during tumorigenesis in LS patients.

#### Somatic Mutational Profile in LS Tumors

Using NGS, Sekine and colleagues investigated the genetic profile of colonic lesions from 44 LS patients and 84 sporadic colorectal adenomas. Most adenomas and all the adenocarcinomas from LS patients were MMR-deficient (MMR-D) and characterized by high-grade MSI. On the other hand, all the sporadic adenomas analyzed were MMR-proficient (MMR-P) and microsatellite stable (MSS) [36]. The genetic profile of colonic lesions showed that *RNF43* gene mutations were frequent in both LS-associated adenomas and adenocarcinomas, while *APC* gene mutations, despite being detected in 40% of LS-adenomas, were more frequent in sporadic colorectal adenomas (60% of cases) (Table 1). The authors observed a distinct somatic mutational profile in LS-associated adenomas depending on the MMR status, and supported the concept that MMR-deficiency occurs prior to the formation of adenoma in LS patients. In particular, MMR-D adenomas were characterized by *RNF43* frameshift somatic mutations in mononucleotide repeats, frequently associated with insertions or deletions of three-repeat sequences in the *APC* gene. By contrast, MMR-P adenomas had a higher frequency of *APC* and *CTNNB1* somatic mutations, but no *RNF43* somatic mutations [36] (Table 1). Otherwise, other studies provided evidence of a late MMR inactivation in LS since the loss of MMR proteins has mainly been found in adenomas with high-grade dysplasia [37,48].

The mutually-exclusive relationship between *RNF43* and *CTNNB1* mutations in LS tumors was confirmed in another study which defined two subgroups of LS-CRCs (G1 and G2), characterized by different amounts and patterns of somatic mutations and microsatellite (MS) slippage [38]. The G1 LS-CRCs subgroup, characterized by the higher amount of mutations and MS slippage, was associated with *MLH1* impairment and somatic mutations in *KRAS*, *TP53*, *POLE*, *MSH3*, *ACVR2A*, *TGFBR2*, *CDC27*, *AIM2,* and *PDS5B* genes. However, the G2 subgroup had a lower degree of MS instability and mutation rate [38] (Table 1). In addition, a transcriptomic analysis of LS adenomas showed increased expression of colonic stem cell markers *CD44*, *BIRC5*, *CCND1*, *MYC* and *ASCL2*, which contribute to sustaining stem cells proliferation in colonic crypts. In particular, *MYC* induction in the G1 LS-CRCs subgroup, seems to be mainly associated with somatic mutations in *ACVRA*, *TCF7LA,* and *TGFBR2* genes [38].

When DNA methylation changes and somatic mutations in formalin-fixed and paraffin-embedded tissues from 57 LS patients were investigated, targeted-NGS showed that somatic mutations in LS-adenomas frequently affect *CTNNB1, SMAD4, KRAS,* and *TP53* genes, with a frequency from 15% up to 24% respectively. In addition, a higher mutation rate was in MMR-D adenomas than in MMR-P lesions [37] (Table 1).

Recent findings have shown distinct associations between MMR mutations and cancer risks [49,50] (Supplementary Table S1). A prospective study demonstrated that carriers of *MSH2* mutations show the highest risk of developing adenomas and advanced adenomas, probably as a consequence of the association between germline *MSH2* alterations and somatic *APC* gene mutations which may contribute to accelerate the colonic malignant transformation [49]. Although carriers of *MSH6* germline mutations, who exhibit a lower amount of MMR-D adenomas, had a great incidence of adenomas, patients with *MSH2* and *MLH1* germline mutations showed the highest risk of early CRC onset [36,49,51] (Supplementary Table S1). In addition, patients with *PMS2* germline mutations had a lower CRC risk, a high frequency of MMR-P adenomas and were negative for *CTNNB1* somatic mutations. On the other hand, *CTNNB1* somatic mutations were frequent in *MLH1*-mutant CRCs [52] (Supplementary Table S1).

The correlation between *CTNNB1* somatic mutations and *MLH1* germline mutations was confirmed in another study, which suggested that *MLH1*-mutant cancers may develop from MMR-deficient crypt foci (MMR-DCF) [49]. MMR-DCF have been described as a new LS-associated lesions, displaying a loss of MMR protein expression, MSI and a distinct non-adenomatous phenotype associated with a rapid invasive growth. In this setting, *CTNNB1* somatic mutations associated with MMR deficiency may possibly act as driver events for LS cancer progression [53].

Based on the concept that MMR-deficiency may occur both as early or late events, three different LS CRC pathways were recently proposed [54]. In fact, according to this hypothesis, LS-associated CRCs may develop following the expansion of adenomas in a MMR-P setting, in which the loss of functional MMR genes is a later event (23% of LS adenomas). Most LS CRCs are associated with early MMR proteins loss and would develop from MMR-D adenomas or MMR-DCF [54]. Importantly, LS-CRCs derived from MMR-DCF represent only 3.3% of cases [54]. Ahadova and colleagues found that somatic mutations in *APC* and *KRAS* genes were mainly associated with MMR-D adenomas in LS patients. The occurrence of these mutations was predicted as a secondary event following MMR-deficiency in MMR-D adenomas. *CTNNB1* and *TP53* somatic mutations have been described as an early event in MMR-DCF [54]. In fact, *TP53* somatic mutations have also been identified in LS-associated adenomas.

Interestingly, most *TP53* somatic mutations have been found in MMR-P adenomas or low-grade dysplastic adenomas. Somatic *KRAS* mutations (G12V and A146T) have also been observed above all in MMR-P adenomas, rather than MMR-D [37,38].

## 3. Sporadic Colorectal Adenomas

### 3.1. Conventional Colorectal Adenomas

Most CRCs arising from CNADs develop following the canonical adenoma-carcinoma sequence, characterized by the accumulation, through a multi-step process, of mutational events in both driver and passenger genes [2].

In 80–90% of CRC cases, the initiating mutational event arises in the *APC* tumor suppressor gene. The subsequent mutational events mainly affect the *KRAS/NRAS* and *TP53* genes. *TP53* mutations are also traditionally associated with *TGF-β*, *SMAD4* and *PI3KCA* mutations during the later phases of adenoma-carcinoma transition [55]. Despite being more frequent in the advanced stages of colorectal tumorigenesis, there is also evidence of *TP53* mutations in normal colon epithelial stem cells [56], as well as in early and premalignant colorectal adenomas [57,58,59].

Although genetic alterations in CRC are well characterized, the genetic and molecular events, which take place during the early stages of tumorigenesis, remain largely unknown. The accumulation of somatic mutations during the adenoma-carcinoma transition is crucial for CRC development [56,60,61]. In this context, the diffusion of NGS techniques has increasingly led to enhancements in understanding the complexity of the tumor mutational landscape [62]. Table 2 summarizes the frequent mutated and new driver genes found in both conventional and serrated adenomas in recent studies.

Using target-NGS, Sievers and colleagues analyzed the genetic profile of 48 small colorectal polyps (6–9 mm) from 36 patients including conventional, serrated and hyperplastic adenomas. As expected, *APC* gene mutations represented the most frequent genetic event (67% of all polyps). In addition pathogenic mutations in *KRAS, FBXW7* and *TP53* genes were also detected in 15%, 10%, and 8% of all the analyzed polyps, respectively [57] (Table 2). *BRAF* p.V600E mutations were also detected in small colorectal polyps, above all in SSAs and HPs. Interestingly, a small percentage of these polyps were characterized by the simultaneous presence of pathological mutations in different driver genes including *APC*, *KRAS*, *TP53* and *FBXW7* [57]. These results support the “Big Bang” model of colorectal tumor development according to which different sub-clones are generated during the tumor growth leading to ITH [76].

By performing single-cell WES and bulk WES on both adenomas and carcinomas from two patients, Wu and colleagues described new early driver alterations in CRC. A mutation in the *OR1B1* gene was described as an early causative event for adenoma development. Mutations in *CSMD1*, *FBXO15*, and *TFAP2D* genes were also identified as sub-clonal mutations contributing to the ITH [63] (Table 2).

Through WES and target sequencing, another study evaluated somatically mutated genes in both CNADs (n = 135) and SSAs (n = 14). In CNADs, *APC* gene mutations represented the most frequent causative event. *KRTAP4-5* (rs411367), *CTNNB1* (rs121913409), *GOLGA8B* (rs200544945), and *TMPRSS13* (rs61900347), *KRAS* (rs1291913529, rs121913530) were also proposed as driver oncogenic mutations in CNADs [60]. In addition, mutations in the tumor suppressor genes *FBXW7* and *SOX9* were found to be driver events in CNADs [60] (Table 2).

In line with these results, a study on 11 colorectal adenoma-carcinoma pairs, found that alterations in *TCF7L2* and *TMPRSS13* genes also contributed to CRC initiation. Interestingly, the authors found two *TCF7L2*-adenoma-specific mutations [59] (Table 2). They also found recurrent mutations in the *NRAS* gene, despite being reported with a low frequency in another study [59,64].

The pivotal role of alterations in the Wnt-pathway related genes in colorectal tumorigenesis was also supported in a retrospective analysis of 58 CNADs with different grades of dysplasia, 17 of which were classified as adenocarcinomas [58]. *APC* gene mutations frequently occurred in this study (76.5% of cases). *KRAS* mutations were found in most of the adenomas analyzed (62.4% of cases), particularly in pre-malignant and high-grade dysplasia adenomas. Mutations in other genes, including *BCL2*, *FBXW7*, *GNAS*, *HNF1A*, *MLL2/KMT2D*, *MLL3/KMT2C*, *SYNE1*, *TCF7L2*, *NOTCH1*, *PBRM1*, *RET*, *RARA*, and *FN1,* were also detected [58] (Table 2). Mutations in the Wnt-related genes *CTNNB1*, *EP300*, *TCF7L2*, and the *AMER1* genes were also observed, but at a lower frequency, in accordance with previous data on adenomas with high grade dysplasia [65]. The authors also reported previously undescribed mutations in *MTOR*, *ACVR1B*, *GNAQ*, *ATM*, *CNOT1*, *EP300*, *ARID2*, *RET*, and *MAP2K4* genes have been reported in colonic adenomas in this study [65] (Table 2).

Another study has helped to establishing which alterations, early identifiable in adenomatous tissues, may play a critical role in CRC development. The authors evaluated the genetic and molecular characteristics of multiple adenomatous tissues from 38 patients through WES and RNA sequencing. They analyzed the differences between cancer-adjacent polyps (CAPs) and cancer-free polyps (CFPs) and found a higher mutation rate in the first group. Interestingly, while both CAPs and CFPs shared causative mutations in the *APC* gene, somatic mutations in *TP53*, *FBXW7*, *PIK3CA*, *KIAA1804*, and *SMAD2* were detected only in CAPs and were related to cancer progression [66] (Table 2).

### 3.2. Colorectal Serrated Lesions

Through an exome-sequencing based approach on peripheral blood or mouthwash samples, the first study to investigate the hereditary genetic alterations associated with the development of SSLs was conducted by Gala and colleagues on 20 unrelated patients with multiple SSLs, most of which met the clinical criteria for SPS. The authors found that loss of function germline mutations in genes involved in the regulation of senescence, particularly *ATM*, *PIF1*, *TELO2*, *XAF1,* and *RBL1*, led to the genetic predisposition of developing multiple SSLs. In addition, this study was the first to identified *RNF43* R113X germline mutation as a causative driver gene for SPS onset. Genotyping one representative SSL from 19 individuals of the 20 enrolled patients, the authors found out that all the lesions, except one, carried the somatic *BRAFV600E* mutation [67] (Table 2).

The *RNF43* gene encodes for an E3 ubiquitin-protein ligase implicated in the ubiquitination and internalization of the Frizzled receptors, thus representing a crucial negative regulator of the Wnt/β-catenin signaling pathway [77,78,79].

After the first evidence suggesting the causative role of germline inactivating mutations in the *RNF43* gene on SPS onset, another study confirmed the pathogenic role of hereditary *RNF43* mutations in one SPS family and further demonstrated that somatic mutations in the *RNF43* gene represent also a frequent event in SSLs (34%), despite not being found in HPs. Importantly, in most sporadic SSLs/TSAs analyzed in this study, the authors found co-occurrent mutations in both *RNF43* and *BRAF* genes (the latter were found only in SSLs/TSAs but not in CNADs) [68] (Table 2). Similar results were reported by Tsai and colleagues. In agreement with other studies, they observed that *RNF43* mutations in TSAs are frequently associated with *BRAF* mutations (14/17 TSAs), rather than *KRAS* mutations (2/17 TSAs) [69] (Table 2).

Although *RNF43* loss of function mutations have been found in both SSLs and TSAs, different studies have shown a higher frequency of somatic *RNF43* mutations in TSAs rather than SSLs [69,70] (Table 2). The presence of mutations in Wnt-related genes was investigated in another study in a series of SSLs with or without dysplasia. The authors found that *RNF43* inactivating mutations constitute the most frequent event in dysplastic lesions, while *APC* and *ZNRF3* alterations were only observed in a few cases (Table 2). Alterations in Wnt signaling components and the consequent β-catenin nuclear accumulation were characteristic of dysplastic lesions, while *BRAF* mutations, found almost in all characterized lesions, did not depend on whether there was dysplasia or not [71].

Performing WES on both CNADs and SSLs, Lin and colleagues found no differences in the mutation frequencies between these adenoma subtypes. However, the mutational profile of CNADs and SSLs was different. The most frequent somatic mutations found in SSLs were *BRAF* (V600E; rs113488022) and *KRTAP4-5* (rs411367). Somatic mutations in the *KRTAP4-5* were found in both CNADs and SSLs, while *APC* gene mutations, observed in most CNADs, were not found in the SSLs analyzed [60] (Table 2). In fact, *APC* gene mutations were found in another study to be more common in CNADs and completely absent in HPs and SSLs, although *APC* mutations were observed in a small fraction of TSAs [70] (Table 2).

The *BRAF* V600E missense somatic mutation was observed in the 67% of cases in a study that prospectively analyzed 200 TSAs. In this study, *BRAF*-mutated adenomas, while having a MSS profile, frequently showed a CIMP-high profile [72] (Table 2). Recently, another study characterized the genetic and molecular profile of a small SSL series with dysplasia or carcinoma showing β-catenin nuclear accumulation in all the analyzed lesions, nearly all of which had *BRAF* mutations. By performing target sequencing, the authors found that about half of the lesions also had mutations in *FBXW7*, particularly MSI-H SSLs, while MSS SSLs partially harbored mutations in the *TP53* gene. Somatic mutations in *KIT*, *PTEN*, *SMAD4,* and *SMARC1B* genes were also detected at low frequencies in the same case series [73] (Table 2).

*APC* gene inactivating mutations represent the most common initiating event during the development of CNADs [2,80]. In an evaluation of the frequency of *APC* gene alterations in the serrated pathway, compared with CNADs, truncating *APC* gene mutations occur over all at a low frequency in serrated lesions, particularly in SSLs, despite their higher rate in TSAs (Table 2). *APC* gene missense mutations have been reported more frequently in MSI-*BRAF*-mutant serrated CRCs (Supplementary Table S2). Despite the low frequency of *APC* gene mutations, increased levels of nuclear β-catenin have been found in both dysplastic SSLs and TSAs, indicating that the hyperactivation of the Wnt/β-catenin pathway might contribute to the malignant progression of serrated lesions with a mechanism that is probably not tied to the *APC* gene inactivation [74].

The *R-Spondin* (*RSPO*) gene fusions, leading to RSPO protein induction, represent an alternative mechanism leading to Wnt/β-catenin signaling induction in TSAs. *RSPO* genes encode for leucine-rich repeat-containing G-protein coupled receptor (LGR) ligands and constitutes potent Wnt agonists, which impair the internalization of Frizzled receptors mediated by *RNF43* and *ZNRF3* [78,81]. *RSPO* fusions have been proposed as drivers in human CRC [82], and have been detected as causative genetic alterations in TSAs [68,70]. Sekine and colleagues found that *PTPRK-RSPO3* fusions were the most frequent cause of *RSPO* overexpression in this setting [70,83]. In addition to the known and most representative *RSPO* fusion transcripts, *PTPRK*(exon 1)-*RSPO3* and *PTPRK*(exon 7)-*RSPO3*, the authors revealed novel *RSPO* fusion isoforms involving the fusion of *PTPRK* exon 6 and 13 to *RSPO3* exon 2, respectively, and the fusion of *NRP1*(exon 2/3) to *RSPO2* exon2 [83]. The same authors confirmed previous results in a further TSAs series and identified *EIF3E-RSPO2* and *PIEZO1-RSPO2* fusions in a small percentage of traditional serrated lesions, which concurrently showed *KRAS* mutations [84]. Moreover, the analysis of precursor polyps associated with TSAs showed that the acquisition of genetic alterations in Wnt-related genes (such as *RNF43, APC* and *CTNNB1*) could occur during the transition from precursor polyps to TSAs, which was more frequent in TSAs [75] (Table 2).

This evidence supports the idea that, although the aberrant activation of the MAPK cascade has a pivotal role in the initiation of the serrated CRC pathway [73], Wnt/β-catenin signaling hyperactivation is critical not only in the malignant progression of CNADs following the classical adenoma-carcinoma sequence, but also in the serrated pathway.

## 4. Conclusions

The main genetic, epigenetic, and molecular alterations driving the onset and progression of CRC in both hereditary and sporadic settings have frequently been investigated. The diffusion of NGS and transcriptomic technologies has led to the identification of new candidate driver genes and to improving the knowledge on the molecular mechanisms underlying the progression of colonic adenomas towards CRC development.

**Figure 1:**
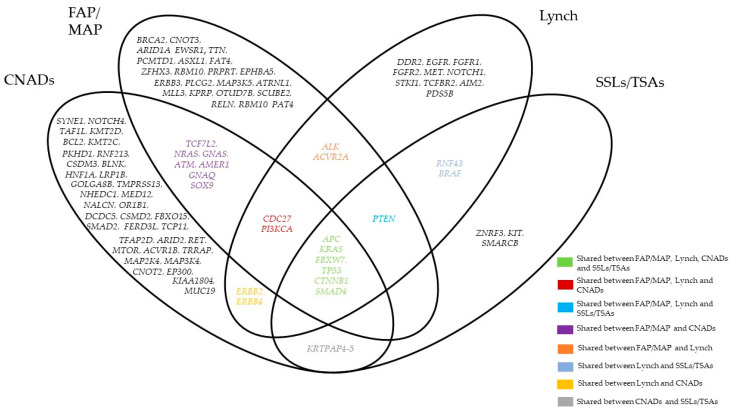
Graphical representation of the most frequent mutated genes in FAP (Familial Adenomatous Polyposis)/MAP (MUTYH-associated Polyposis), Lynch, CNADs (Conventional Colorectal Adenomas) and SSLs (Sessile Serrated Lesions) /TSAs (Traditional Serrated Lesions emerging from the studies discussed in this review. The graph does not refer to any statistics.

Figure 1 summarizes the most common mutated genes in both hereditary and sporadic settings emerging from the studies discussed in this review. Although the mutations of a few genes were shared among these different settings, the studies highlight the wide genetic heterogeneity of both early and advanced pre-cancerous lesions, thus making it difficult to develop effective therapeutic strategies.

Several studies have investigated the long-term CRC risk based on the endoscopic and histological characteristics of colonic lesions. However, studies on large series, simultaneously assessing the risk of developing CRC in relation to the histopathologic, genetic, and molecular characteristics of pre-cancerous lesions, are lacking.

In conclusion, the evaluation of the CRC risk based on the molecular characterization of early pre-cancerous colorectal lesions, both in hereditary and sporadic settings, may speed up the development of targeted preventive strategies. This would consequently define specific risk profiles, as well as identify those patients who would most benefit from strict endoscopic surveillance. Further studies will be pivotal to establish the individual CRC risk according to the genetic alterations detectable in early colorectal lesions and to define the impact of such characterization in clinical practice.

## Figures and Tables

**Table 1:** Studies evaluating somatic mutations in colorectal adenomas and cancers from patients with hereditary CRC syndromes.

Syndrome	Study Population	Samples Analyzed	Study Methods	Frequent Mutated Genes and Candidate Driver Genes	Notes	References
FAP	12 FAP patients	25 adenomas, 10 adjacent NM	WES	*APC, KRAS, FBXW7, TCF7L2, BRCA2, ALK, CNOT3, ARID1A, CDC27, EWSR1, GNAQ, TTN, PCMTD1*		[32]
FAP	14 FAP patients	37 adenomas and matched NM	Targeted Ampliseq sequencing	*APC, KRAS*		[33]
FAP and MAP	5 FAP patients 1 MAP patient	20 LGD adenomas, 4 HGD adenomas, 7 carcinomas and 8 adjacent NM	WES, WGS	*APC* (69%), *TTN* (37%), *SMAD4* (35%), *GNAS* (33%), *ASXL1* (33%), *KRAS* (23%), *FAT4* (22%), *ZFHX3* (22%), *FBXW7* (22%), *PTPRT* (20%), *SOX9* (16%)	Additional potential driver events with lower frequency: *ERBB3* (8%), *ARID1A* (8%), *TP53* (8%), *ACVR2A* (6%), *EPHA5* (6%), *TCF7L2* (6%), *PIK3CA* (6%), *RBM10* (5%), *CTNNB1* (5%), *ATM* (5%), *AMER1* (5%).	[34]
FAP and MAP	2 FAP patients and 2 MAP patients	6 adenomas 8 adenomas	WES	*APC, KRAS, WTX/FAM123B, SCUBE2, RELN, FBXW7, MLL3, OTUD7B, KPRP, ATRNL1, MAP3K5, NRAS, PLCG2, PTEN, TP53*	Except for *APC*, *WTX* and *KRAS,* few adenomas shared the same set of mutated driver genes	[35]
	3 FAP patients and 4 MAP patients	22 adenomas 33 adenomas	Targeted exome sequencing			
	7 FAP patients and 3 MAP patients	41 adenomas 22 adenomas	*WTX/KRAS* capillary sequencing			
Lynch	44 patients	86 adenomas, 36 adenocarcinomas	Target NGS	*APC* (40% of LS-associated adenomas, 28% of LS-adenocarcinomas and 60% sporadic adenomas); *CTNNB1* (5% LS-adenomas and 10% sporadic adenomas); *RNF43* (52% LS-associated adenomas; 56% LS-adenocarcinomas).	*KRAS*, *BRAF* and *NRAS* mutations uncommon in both LS- and sporadic adenomas	[36]
		84 sporadic adenomas				
Lynch	57 patients	59 adenomas: 16 MMR-P; 43 MMR-D (41 LGD and 18 HGD)	Amplicon-based NGS	*TP53* (24%), *KRAS* (22%), *SMAD4* (19%), *CTNNB1* (15%)	Additional potential driver events with lower frequency: *ALK* (2%)*, BRAF* (9%), *DDR2* (2%)*, EGFR* (10%), *ERBB2* (3%)*, ERBB4* (5%), *FBXW7* (9%), *FGFR1* (2%), *FGFR2* (3%), *MET* (2%), *NOTCH1* (5%), *PTEN* (10%), *PIK3CA* (5%), *STKI1* (3%)	
						[37]
Lynch	11 patients	Paired tumor (adenoma and cancer) and tumor-distant NM	Whole- genome DNA- sequencing	*ACVR2A, TGFBR2, CDC27, AIM2, PDS5B, TP53, KRAS* (Frequent in the G1 LS-CRC subgroup)	Paired patient-matched specimens of tumors were stratified into two subgroups based on their genomic characteristics (G1 with higher amount of mutation and MS slippage than G2)	[38]

FAP (Familial Adenomatous Polyposis); MAP (MUTYH-associated Polyposis); NM (Normal Mucosa); LGD (Low-Grade Dysplasia); HGD (High-Grade Dysplasia); WES (Whole-Exome Sequencing); WGS (Whole-Genome Sequencing); NGS (Next-Generation Sequencing); MMR-P (MMR-proficient); MMR-D (MMR-deficient); MS (microsatellite). The frequency of mutations (%) is shown for those studies that have reported them.

**Table 2:** Studies evaluating somatic mutations in sporadic conventional and serrated colorectal lesions.

Study Population	Samples Analyzed	Study Methods	Frequent Mutated Genes and Candidate Driver Genes	Notes	Reference
36 patients	48 colorectal polyps: 33 tubular adenomas, 5 tubulovillous adenomas, 4 SSLs, 6 HPs	Target NGS	*APC* (67%), *KRAS (*15%), *NRAS* (2%), *TP53* (8%), *FBXW7* (10%)*, BRAFV600E* (17%, mainly in SSLs and HPs)		[57]
58 patients with colorectal adenomas (retrospective study)	85 samples from 58 adenomas ≥ 2 cm: 19 LGD adenomas (10 tubular and 9 tubulovillous adenomas); 21 premalignant adenomas; 28 HGD adenomas; and 17 invasive adenocarcinomas	Target NGS	*APC* (76.5%)*, KRAS* (62.4%)*, SYNE1* (35.3%)*, NOTCH4* (23.5%), *TCF7L2* (18.8%), *GNAS* (17.6%), *FBXW7* (15.3%), *TAF1L* (15.3%), *KMT2D* (15.33%) *BCL2* (12.9%), *KMT2C* (11.8%), *PKHD1* (11.8%*), RNF213* (10.6%), *CSDM3* (10.6%), *TP53* (20%), *BLNK* (17.6%), *HNF1A* (12.9%), *LRP1B* (10.6%)	The percentages refer to all the analyzed adenomatous samples. Data on cancer samples are described in Supplementary Table S2	[58]
NA	149 adenomas from two independent projects: 100 CNADs (first study); 35 CNADs and 14 SSLs (second study)	WES and Target sequencing	CNADs: *APC, CTNNB1, KRTAP4-5, GOLGA8B, TMPRSS13, TP53, NHEDC1, PI3KCA, KRAS, FBXW7, SOX9, ATM, CDC27, MED12, NALCN;* SSLs: *KRTAP4-5, BRAFV600E*		[60]
2 CRC patients	24 NM single cells; 48 adenoma single cells (tubular adenomatous polyps and inflammatory fibroid polyp normal appearance)	single-cell WES and bulk WES	*OR1B1, DCDC5, CSMD1, FBXO15, TCP11, TFAP2D*	Small sample size. Data on CRC single cells are described in Supplementary Table S2	[63]
NA	11 colorectal adenoma-carcinoma pairs	WES	*APC, CTNNB1, KRAS, TP53, TMPRSS13, TFC7L2, NRAS, FERD3L*	Data on gastric cancer not included. Data on cancer samples are described in Supplementary Table S2	[59]
2 large prospective cohort studies: Nurses’ Health Study (N = 121,700 women followed since 1976) and Health Professionals Follow-up Study (N = 51,500 men followed since 1986)	225 colorectal cancers LGD (=50% gland formation) vs. HGD (<50% gland formation)	Pyrosequencing and Sanger sequencing	*NRAS* activating mutations (5/225 CNADs c.34G>A, c.35G>A and c.35G>T in codon 12 and c.181C>A in codon 61)	Only *NRAS* mutations were analyzed in this study	[64]
12 Korean patients	12 high-grade colon adenoma samples (11 non-hypermutated and 1 hypermutated (POLE-mutated) tubulovillous adenomas) and matched NM	WES	*APC, KRAS, SMAD4, ERBB4, TCF7L2, AMER1, TP53, GNAS, ARID2, RET, MTOR, NRAS, ACVR1B, GNAQ, ATM, PIK3CA, ERBB2, TRRAP, MAP2K4, MAP3K4, CNOT2, EP300*		[65]
31 patients	90 tissues: 16 CAP cases matched with 15 CFP cases-all polyps are adenomatous polyps with villous features -tubulovillous or villous- and LGD)	WES	*TP53, FBXW7, PIK3CA, KIAA1804, SMAD2* and SMAD4 (mutations exclusively in CAP samples); *APC* (significantly mutated in both polyp groups, 70% CFPs and 80% CAPs); *MUC19* (CFPs)		[66]
20 patients with multiple SSLs (16 fulfill WHO clinical criteria for SPS)	1 SSL from each individual (19/20)	*BRAF/* *KRAS* SNaPshot genotyping	*BRAFV600E* (18/19 SSLs)		[67]
5 patients(carriers of *RNF43* c.953-1, G>A germline mutation) from 1 SPS family	16 serrated lesions (SSLs/TSAs/HPs), 5 tubulovillous/ villous adenomas and 1 cancer (from 5 SPS patients); 90 sporadic lesions (14 HPs, 47 SSLs/TSAs, 29 CNADs);	WES, Target Gene Sanger Sequencing	*RNF43* (18.8% serrated lesions from SPS patients; 20% conventional adenomas from SPS patients; 34% sporadic SSLs/TSAs; 0% sporadic HPs; 3.4% sporadic CNADs); *BRAFV600E* (62.5% SSLs/TSAs from SPS patients; 66% sporadic SSLs/TSAs; 81.2% sporadic SSLs/TSAs *RNF43*-mutated)	SSLs and TSAs analyzed as a single group. Data on cancer samples are described in Supplementary Table S2	[68]
NA	20 SSLs; 36 TSAs; 37 TSAs with cytologic dysplasia; 30 tubulovillous/ villous adenomas	Sanger sequencing	*RNF43* (10% SSLs; 28% TSAs; 19% TSAs with dysplasia; 0% tubulovillous/villous adenomas), *BRAF* (82% TSAs *RNF43*- mutated with/without dysplasia), *KRAS* (11.7% TSAs *RNF43*- mutated with/without dysplasia)	Data on cancer samples are reported in Supplementary Table S2	[69]
NA	130 serrated lesions (26 HPs, 34 SSLs, 70 TSAs) and 58 CNADs (27 tubular adenomas, 31 tubulovillous adenomas)	Target NGS, Sanger sequencing	*RNF43* (6% SSLs; 24% TSAs; 0% CNADs); *BRAF* (73% HPs; 74% SSLs; 60% TSAs; 0% CNADs); *APC* (0% HPs and SSLs; 13% TSAs; 59% CNADs)		[70]
NA	46 dysplastic SSLs; 45 SSLs without dysplasia	Target NGS, Sanger sequencing	*RNF43* (50% dysplastic SSLs); *APC* (9% dysplastic SSLs), *ZNRF3* (7% dysplastic SSLs); *BRAF* (87% dysplastic SSLs; 84% SSLs without dysplasia)		[71]
196 patients	200 TSAs (162 ordinary, 38 advanced); 50 tubulovillous adenomas	Allele Specific PCR	*BRAFV600E* (67% TSAs)		[72]
8 patients	8 SSLs: 4 SSLs with HGD, 4 SSLs with submucosal carcinoma	Target NGS	*BRAFV600E* (88% SSLs); *FBXW7* (38% SSLs); *TP53* (25% SSLs); *KIT, PTEN, SMAD4, SMARCB* (13% SSLs each)		[73]
NA	189 samples: 20 SSLs; 20 dysplastic SSLs; 14 TSAs; 6 dysplastic TSAs; 19 tubular and tubulovillous adenomas	Targeted amplicon sequencing	*APC* (5% SSLs; 20% dysplastic SSLs; 36% TSAs; 33% dysplastic TSAs; 89% tubular and tubulovillous adenomas)	Only *APC* mutations were analyzed in this study. Data on cancer samples are reported in Supplementary Table S2	[74]
NA	15 TSAs associated with precursors polyps: 9 associated with HPs and 6 associated with SSLs	Laser microdissection based sequencing	*RNF43, APC, CTNNB1; BRAFV600E*		[75]

CNADs (Conventional Colorectal Adenomas); SSLs (Sessile Serrated Lesions); TSAs (Traditional Serrated Adenomas); HP (Hyperplastic Polyps); CAPs (Cancer-Adjacent Polyps); CFPs (Cancer-Free Polyps); NM (Normal Mucosa); LGD (Low-Grade Dysplasia); HGD (High-Grade Dysplasia); WES (Whole-Exome Sequencing); NGS (Next-Generation Sequencing); NA (Not Available). The frequency of mutations (%) is shown for those studies that have reported them.

## Supplementary Materials

The following are available online at https://www.mdpi.com/article/10.3390/cancers13092081/s1, Table S1: Studies evaluating cumulative CRC risk in Lynch syndrome in relation to germline mutations in MMR gene; Table S2: Studies evaluating somatic mutations in sporadic CRC. References [49,50,51,52,58,59,63,68,69,74] are cited in the Supplementary Material.
