# Supplementary material for: Discovering the Mutational Profile of Early Colorectal Lesions: A Translational Impact
Source: Cancers (Basel). 2021 Apr 25;13(9):2081. doi: 10.3390/cancers13092081 (PMC8123354; doi:10.3390/cancers13092081)
Supplement: Supplementary file 1 [file cancers-13-02081-s001.zip › cancers-1190138-supplementary.pdf]

Supplementary Materials

Discovering the Mutational Profile of Early Colorectal Lesions: A Translational Impact

Chiara Alquati, Anna Prossomariti, Giulia Piazzi, Francesco Buttitta, Franco Bazzoli, Luigi Laghi and Luigi Ricciardiello

Table S1. Studies evaluating cumulative CRC risk in Lynch syndrome in relation to germline mutations in MMR gene.

Study Population	Germline Mutation	Associated Somatic Mutations	Median Follow-up period	Cumulative CRC risk	Notes	References
29 patients	*MSH2*	*APC* (75%); *CTNNB1* (7%)	7.8 years	11.4%	Small sample size for *MSH6* germline mutation	[49]
16 patients	*MLH1*	*APC* (11%); *CTNNB1* (50%)		11.3%		
3 patients	*MSH6*	*APC* (100%)		4.7%		
159 patients	*MSH2*	NA	6.5 years	17.8%	Small sample size for *PMS2* germline mutation. The percentages refer to the cumulative risk of CRC by 70 years of age	[50]
98 patients	*MLH1*	NA		17.7%		
103 patients	*MSH6*	NA		8.5%		
21 patients	*PMS2*	NA		0%		
1060 patients	*MSH2*	NA	NA	43%*	The study evaluated the **cumulative incidence (*)** of CRC at 75 years of age. The study analyzed also endometrial, ovarian, urinary tract, prostate, brain and upper gastrointestinal cancers.	[51]
1473 patients	*MLH1*	NA		46%*		
462 patients	*MSH6*	NA		15%*		
124 patients	*PMS2*	NA		0%*		
18 patients	*MSH2*	*CTNNB1* 6%; *KRAS* 39%; *PIK3CA* 39%; *APC* 33%; *TP53* 28%; *FBXW7* 17%;	NA	NA		[52]
24 patients	*MLH1*	*CTNNB1* 58%; *KRAS* 29%; *PIK3CA* 25%; *APC* 13%; *TP53* 25%; *FBXW7* 17%				
24 patients	*PMS2*	*CTNNB1* 0%; *KRAS* 50%; *PIK3CA* 25%; *APC* 30%; *TP53* 25%; *FBXW7* 20%;				

NA (Not Available); * data refer to cumulative incidence.

Table S2. Studies evaluating somatic mutations in sporadic CRC.

Study population	Samples analyzed	Study Methods	Frequent mutated genes and candidate driver genes	Notes	References
58 patients with colorectal adenomas (Retrospective study)	17 invasive adenocarcinomas	Target NGS	*APC* (76.5%), *KRAS* (41.1%), *SYNE1* (47.1%), *NOTCH4 (17.6%)*, *TCF7L2* (17.6%), *GNAS* (5.9%), *FBXW7* (5.9%), *TAF1L* (17.6%), *KMT2D* (17.6%), *BCL2* (29.4%), *KMT2C* (17.6%), *PKHD1* (5.9%), *RNF213* (5.9%), *CSDM3* (5.9%), *TP53* (35.3%), *BLNK* (23.5%), *HNF1A* (0%), *LRP1B* (17.6%)	The percentages refer to adenocarcinomas	[58]
2 CRC patients	96 single cells from CRC	single-cell WES and bulk WES	*LAMA1, ADCY3*	Small sample size	[64]
NA	11 colorectal adenoma-carcinoma pairs	WES	*APC, CTNNB1, FERD3L, KRAS, TP53, TMPRSS13, NRAS, KRTAP5-1, OR2T35, FOXC1*	Data on gastric cancer not included	[59]
NA	47 MSI CRCs and 64 MSI/*MMR*-mutated CRCs from Hong Kong cohort; 57 TCGA MSI CRCs; 391 TCGA MSS CRCs; 137 Giannakis MSS CRCs	WES, Target Gene Sanger Sequencing	*RNF43* (79.6% MSI CRCs *MLH1* met; 35.5% MSI CRCs *MLH1* unmet; 3.03% MSS CRCs*); BRAFV600E* (67.79% MSI CRCs *MLH1* met; 4.4% MSI CRCs *MLH1* unmet; 5.94% MSS CRCs); *KRAS* (57.5% MSI CRCs *MLH1* unmet; 11.86% MSI CRCs *MLH1* met); *APC* (50% MSI CRCs *MLH1* unmet; 23% MSI CRCs *MLH1* met); *TP53*(33.3% MSI CRCs *MLH1* unmet; 30.8% MSI CRCs *MLH1* met); *CTNNB1* (20% MSI CRCs *MLH1* unmet; 3.38% MSI CRCs *MLH1* met)	*TP53* and *APC* mutations evaluated in TCGA MSI CRCs cohort only. Differences between MSI and MSS CRCs were not described.	[72]
NA	31 *BRAF*-mutated/MSS carcinomas	Sanger sequencing	*RNF43* (29%)	The study described only the relationship between *BRAF* and *RNF43* mutations	[73]
NA	80 *BRAF*-mutant cancers (50 MSI, 30 MSS); 30 *BRAF* wild-type cancers	Targeted amplicon sequencing	*APC* (70% *BRAF* wild-type cancers; 40% MSI *BRAF*-mutated cancers; 20% MSS *BRAF*-mutated cancers)	Only *APC* mutations were analyzed in this study	[79]

MSI (Microsatellite Instability Pathway); MSS (Microsatellite Stable); WES (Whole-Exome Sequencing); NGS (Next-Generation Sequencing); MLH1 met (MLH1-methylated); MLH1 unmet (MLH1-unmethylated); NA (Not Available). The frequency of mutations (%) is shown for those studies that have reported them.
